# Epidemiology and regional variance of traumatic peripheral nerve injuries in Sweden: A 15-year observational study

**DOI:** 10.1371/journal.pone.0310988

**Published:** 2024-10-09

**Authors:** Martin Magnéli, Michael Axenhus

**Affiliations:** 1 Department of Orthopaedic Surgery, Danderyd Hospital, Stockholm, Sweden; 2 Department of Clinical Sciences, Danderyd Hospital, Stockholm, Sweden; Foundazione Policlinico Universitario Agostino Gemelli, ITALY

## Abstract

**Introduction:**

Traumatic peripheral nerve injuries pose significant challenges to healthcare systems and individuals, affecting sensory function, causing neuropathic pain, and impairing quality of life. Despite their impact, comprehensive studies on the epidemiology and regional variance of these injuries are scarce. Understanding the incidence, trends, and anatomical distribution of such injuries is essential for targeted interventions and resource allocation.

**Methods:**

This observational study utilized register-based data from the Swedish National Patient Register covering the period from 2008 to 2022. Incidence rates, trends, and anatomical distribution of traumatic peripheral nerve injuries were analyzed using descriptive statistics, Poisson regression modeling, and regional comparisons.

**Results:**

Higher incidences of peripheral nerve injuries were observed among men compared to women across all age groups. The hand and wrist were the most commonly affected sites. Regional variations in incidence rates were evident, with some regions consistently exhibiting higher rates compared to others. Notably, a decreasing trend in injuries was observed over the study period.

**Conclusion:**

This study underscores the importance of targeted interventions and preventive strategies, considering sex, age, and regional disparities. Further research incorporating individual patient-level data is warranted to enhance our understanding and inform tailored interventions to reduce the burden of these injuries.

## Introduction

Injuries to peripheral nerves across the body represent a significant issue for affected patients and are associated with comorbidity and decrease in quality of life [1]. In a Swedish population, the reported incidence of such injuries stands at approximately 13.9 per 100,000 individuals [2]. Peripheral nerve injuries can be caused by trauma, complications from systematic disease or be part of neurodegenerative processes [3–5]. Peripheral nerve injuries can manifest as impaired sensory function, neuropathic pain and loss of proception, necessitating substantial healthcare resources [6,7]. However, comprehensive studies delineating the incidence, regional variance, trends, and anatomical distribution of traumatic peripheral nerve injury are lacking. Evidence suggests that early rehabilitation interventions yield favorable outcomes in recovering from major nerve injuries, making it important to identify vulnerable populations to direct healthcare interventions and rehabilitation [8,9]. Rehabilitation can often be started promptly after injury, even before axonal re-innervation of the affected area takes place, focusing on sensory relearning [10,11].

The primary aim of this study is to examine the epidemiology and regional variance of traumatic peripheral nerves injuries in Sweden. Our secondary aim was to identify vulnerable patient population in respect to sex and age.

## Materials and methods

### Ethics approval and consent to participate

The data used in this study is obtained from the website of the NPR and is publicly available for anyone to download and use.

### Study design setting

This study is an observational national-level analysis of registry data on traumatic peripheral nerve injuries in Sweden between 2008 and 2022. Data was obtained from the Swedish National Patient Register (NPR) [12]. Population data was obtained from statistics Sweden [13]. Over the study period, Sweden’s population grew from 9.21 million to 10.49 million inhabitants. The study adheres to the RECORD guidelines [14].

### Setting

Sweden is a high income, northern European country of approximately 10,50 million inhabitants in 2023. Sweden is geographically divided into 21 region who are responsible for certain administrative duties within their defined areas. These duties include public transportation and healthcare. Due to its geographical variation, Swedish regions experience vastly different conditions for the delivery of healthcare with the southern regions experiencing shorter distances and higher population density compared to northern regions.

### Data source

The NPR, established in 1964 for inpatient care and expanded nationwide in 1987, encompasses specialized outpatient care since 2001. It receives monthly updates, ensuring data currency and accuracy. All hospitals and specialized centers, irrespective of ownership, contribute data to the NPR, including diagnosis codes based on the International Statistical Classification of Diseases version 10 (ICD-10) [15]. Reporting to the NPR has been mandatory for reporting on a national scale since 1987. The NPR provides extensive information on all public inpatient care in Sweden, including patient demographics, hospital records, administrative details, and medical data, such as personal identification numbers, sex, age, and area of residence down to the parish level. This mandatory reporting helps to minimize underreporting and ensures a high level of completeness and accuracy.Patient are tracked in the NPR using a personal identification number which is unique to the individual and only discarded upon death or emigration, making it suitable for population level data analysis. The data included in the NPR has been subject to external validation studies, which have confirmed its accuracy and completeness in capturing patient-level healthcare data across Sweden [16]. The NPR undergoes regular updates and systematic checks to maintain data integrity.

We linked the incidence data from the NPR with population data from Statistics Sweden by aligning regional identifiers (i.e., the 21 Swedish regions) and time periods (calendar years). This allowed us to accurately calculate incidence rates per 100,000 person-years by ensuring that the population data corresponded to the same timeframes and regions as the injury data. One potential challenge in data linkage was ensuring consistency between the population data and the incidence data, particularly in cases where there were minor discrepancies in how regions were reported or if there were slight timing mismatches between the updates of the two sources. To address any inconsistencies we used the most current population estimates for each year to ensure accurate incidence calculations.

### Study population

The scope of this study encompasses all individuals aged 15 years or older in Sweden that experienced traumatic injuries to the peripheral nervous system, excluding injuries resulting from infectious diseases, autoimmune disorders, inherited conditions, or other non-traumatic causes.

Inclusion criteria:

1. Individuals residing in Sweden at any time during 1^st^ of January 2008 to 31^st^ of December 2022
2. Individuals who have suffered a peripheral nerve injury as specified by ICD-10 coding.

Exclusion criteraia:

1. Peripheral nerve injury due to infection, autoimmune, inherited or non-traumatic cause.

Each unique personal identification number was tallied once annually, per injury, and per geographical region. Peripheral nerve injuries were identified using ICD-10 classification. Details regarding the included ICD-10 codes are provided. We also included ICD-10 codes for traumatic amputation, which naturally include peripheral nerve injury without being specifically coded as such, at each anatomical level. We did not include ICD-10 codes for nerve injuries that were discover post trauma at a later date such as ICD 10 code T9 (Table 1).

**Table 1:** Included ICD-10 codes of traumatic peripheral nerve injuries and amputations.

ICD-10 code	Injury
**S48.0–9**	Traumatic amputation at the shoulder and upper arm region
**S58.0–9**	Traumatic amputation at the forearm region
**S68.0–9**	Traumatic amputation at the wrist and hand region
**S78.0–9**	Traumatic amputation at the hip and thigh region
**S88.0–9**	Traumatic amputation at the lower leg region
**S98.0–9**	Traumatic amputation at the ankle and foot region
**S44.0–9**	Peripheral nerve injury at the shoulder and upper arm region
**S54.0–9**	Peripheral nerve injury at the forearm region
**S64.0–9**	Peripheral nerve injury at the wrist and hand region
**S74.0–9**	Peripheral nerve injury at the hip and thigh region
**S84.0–9**	Peripheral nerve injury at the lower leg region
**S94.0–9**	Peripheral nerve injury at the ankle and foot region

### Statistics

Descriptive statistics were utilized to present total numbers and percentages. The ratio between the maximum and minimum incidence rates was calculated as a comparative measure. T-tests and chi-square tests were employed to compare the distribution of age and sex. National and regional crude rates for all peripheral nerve injuries and each category of peripheral nerve injury were computed per 100,000 person-years, with age, sex, region, and year population counts as the denominator. Traumatic amputation and peripheral nerve injury ICD-10 codes were analyzed together for each anatomical level. Poisson regression or negative binomial regression models were used to estimate incidence rate ratios (IRRs) and corresponding 95% confidence intervals (CIs) for the annual changes in incidence rate over a 15-year period (2008 to 2022), adjusted for age and sex. The suitability of Poisson regression models was assessed using Pearson’s goodness-of-fit test, with negative binomial regression employed in cases where variation exceeded the Poisson distribution. Models were developed for all peripheral nerve injuries and each category of peripheral nerve injury, generating estimates for both sexes combined and stratified by sex. The included covariables were age-stratified incidence data, type of peripheral nerve injury, regional variance, and sex. Covariates were incorporated directly into the statistical models to control for potential confounding factors. Missing data were handled using multiple imputation. Multiple comparisons were addressed using the Bonferroni correction. Residual analysis were performed to assess the fit of the regression models. Overdispersion was checked using the deviance and Pearson chi-square statistics, and adjustments were made where necessary.Regional peripheral nerve injury rates from 2008 to 2022 were compared using the mean of regions as a reference point, with adjustments made for age and sex. Statistical significance was inferred for p-values less than 0.05. P-values are displayed as exact p-values with an accuracy of All statistical analyses were conducted using SPSS (version 27.0) (IBM, New York, USA).

### Ethics

The study utilized publicly available open-source data and was therefore exempt from ethical review.

## Results

**Fig 1:**
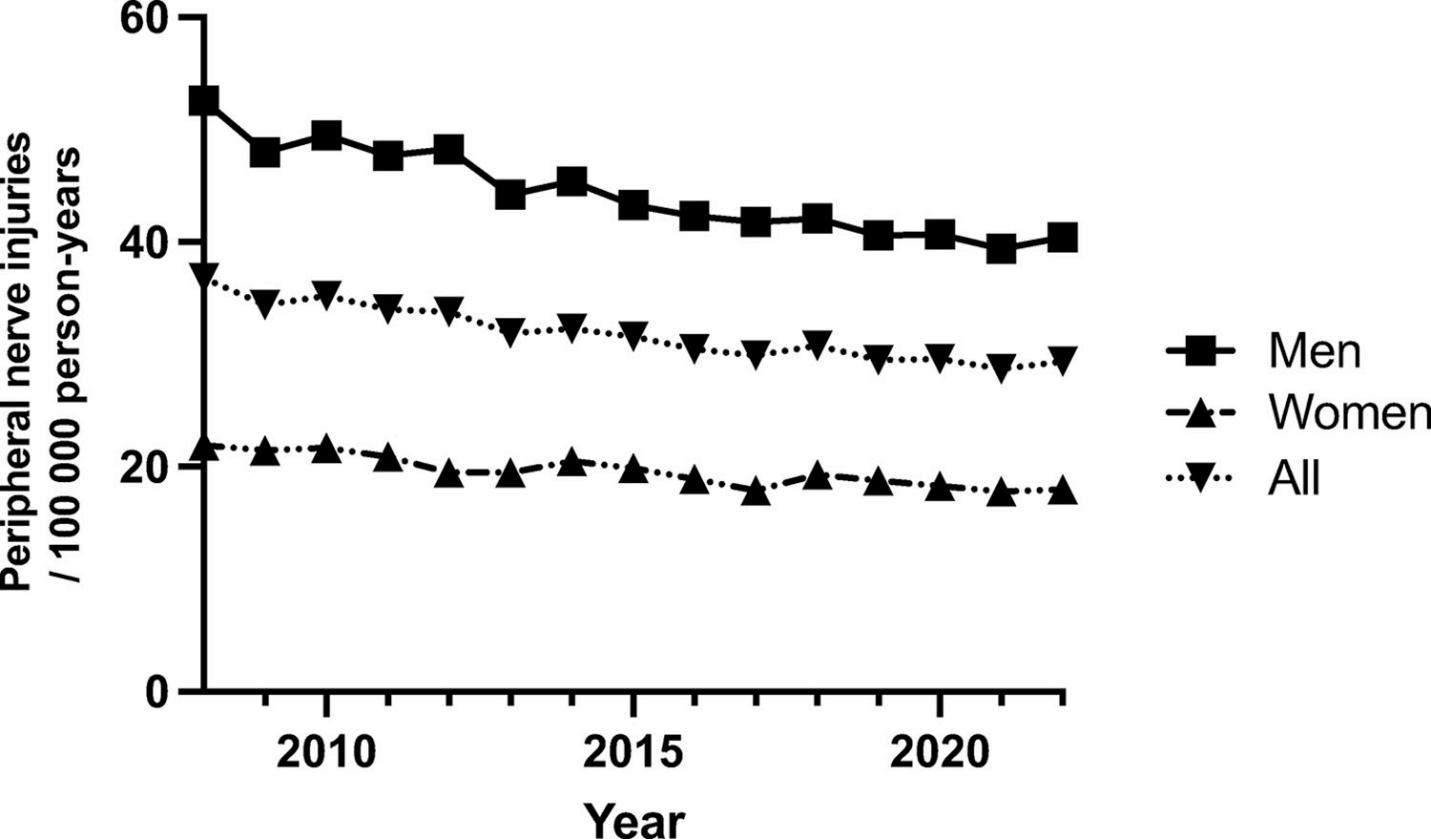
Incidence of peripheral nerve injuries. Incidence of injury amongst men and women during 2008–2022.

The data from 2008 to 2022 indicates a consistent decrease in peripheral nerve injuries among both men and women. There was a significant(p = 0.3 x 10^−3^) difference in the incidence of peripheral nerve injuries between men and women with men experiencing more peripheral nerve injuries than women. Among men, the incidence decreased from 23.1 (95% CI: 20.8–25.4) to 15.6 (95% CI: 13.8–17.4) cases per 100,000 individuals(p = 0.2 x 10^−4^), while among women, it decreased from 14.1(95% CI: 12.8–15.6) to 10.1(95% CI: 9.2–11.0) cases per 100,000 individuals(p = 0.4 x 10^−3^). The overall incidence decreased from 18.6(95% CI: 17.1–20.1) to 12.9(95% CI: 12.1–13.7) cases per 100,000 individuals(p = 0.2 x 10^−4^). The decrease in peripheral nerve injuries was consistent from 2008 to 2013 with a slight uptick during 2014 before dropping again in 2015 and 2016. 2017 saw an uptick in peripheral nerve injuries followed by a notable acceleration in the reduction of injuries in the latter years (Fig 1).

**Fig 2:**
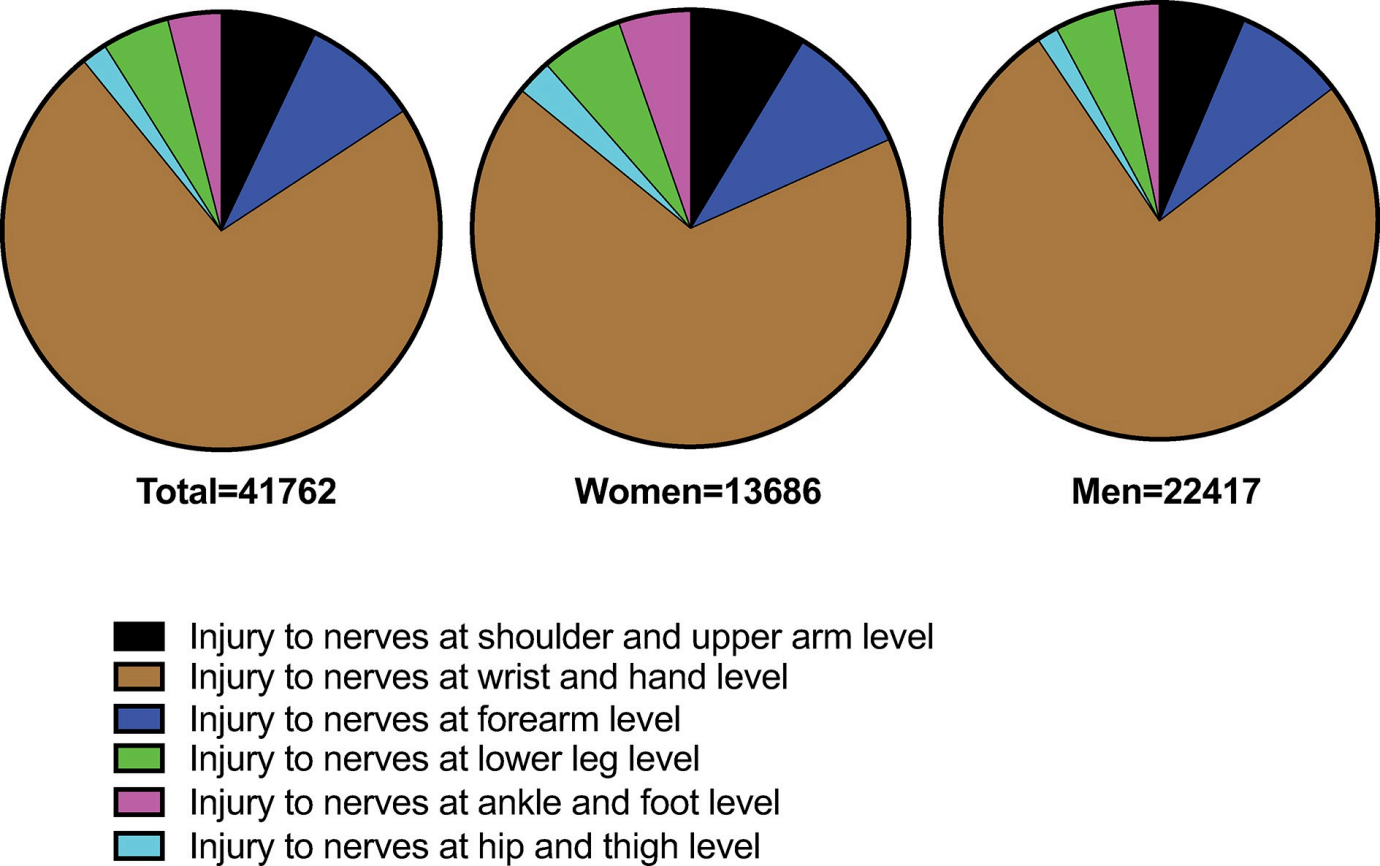
Peripheral nerve injuries and distribution per sex and anatomical location. Colors indicate anatomical location, total injuries include both men and women.

In total, 36 103 cases of injury to peripheral nerves were observed, with 13 686 cases in women and 22 417 in men. Most injuries occurred in the wrist and hand (16,290 cases), followed by the shoulder and upper arm (2,642 cases) and forearm (3,255 cases). The cervical region had 6,162 cases, with 1,951 in women and 4,211 in men. Thoracic injuries were 1,071, with 307 in women and 764 in men. Lumbar injuries were 1,488, with 756 in women and 732 in men. Hip and thigh injuries were 646, with 298 in women and 348 in men. Lower leg injuries were 1,679, with 670 in women and 1,009 in men. Ankle and foot injuries were 1,036, with 515 in women and 521 in men (Fig 2).

**Fig 3:**
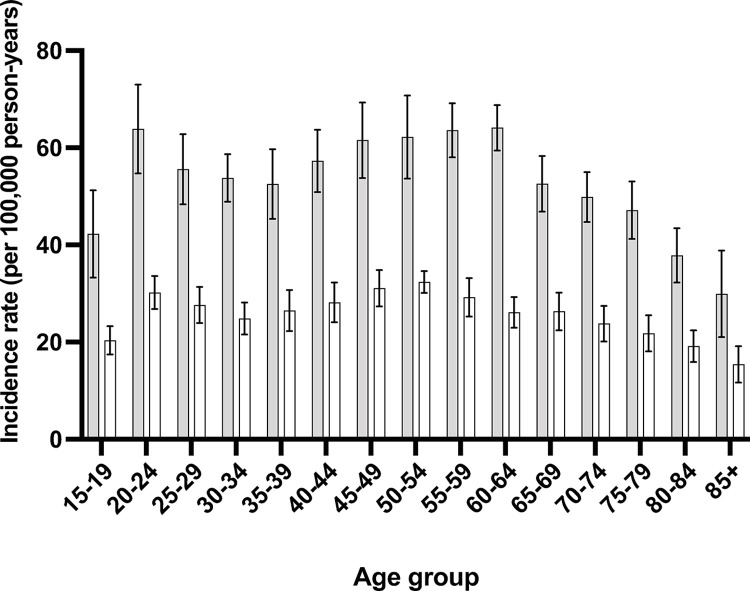
Incidence of peripheral nerve injuries in age and sex groups. White indicates women, grey indicate men. Error bar indicate 95% confidence interval.

Across age groups, males consistently showed higher rates of injuries compared to females (p = 0.7 x 10^−3^). For instance, in the 15–19 age group, males exhibited rates ranging from 25.2% to 72.1%, while females ranged from 14.2% to 35.1%. This trend persisted across all age brackets, with males consistently exhibiting higher percentages of injuries compared to females (p = 0.4 x 10^−3^) (Fig 3).

**Fig 4:**
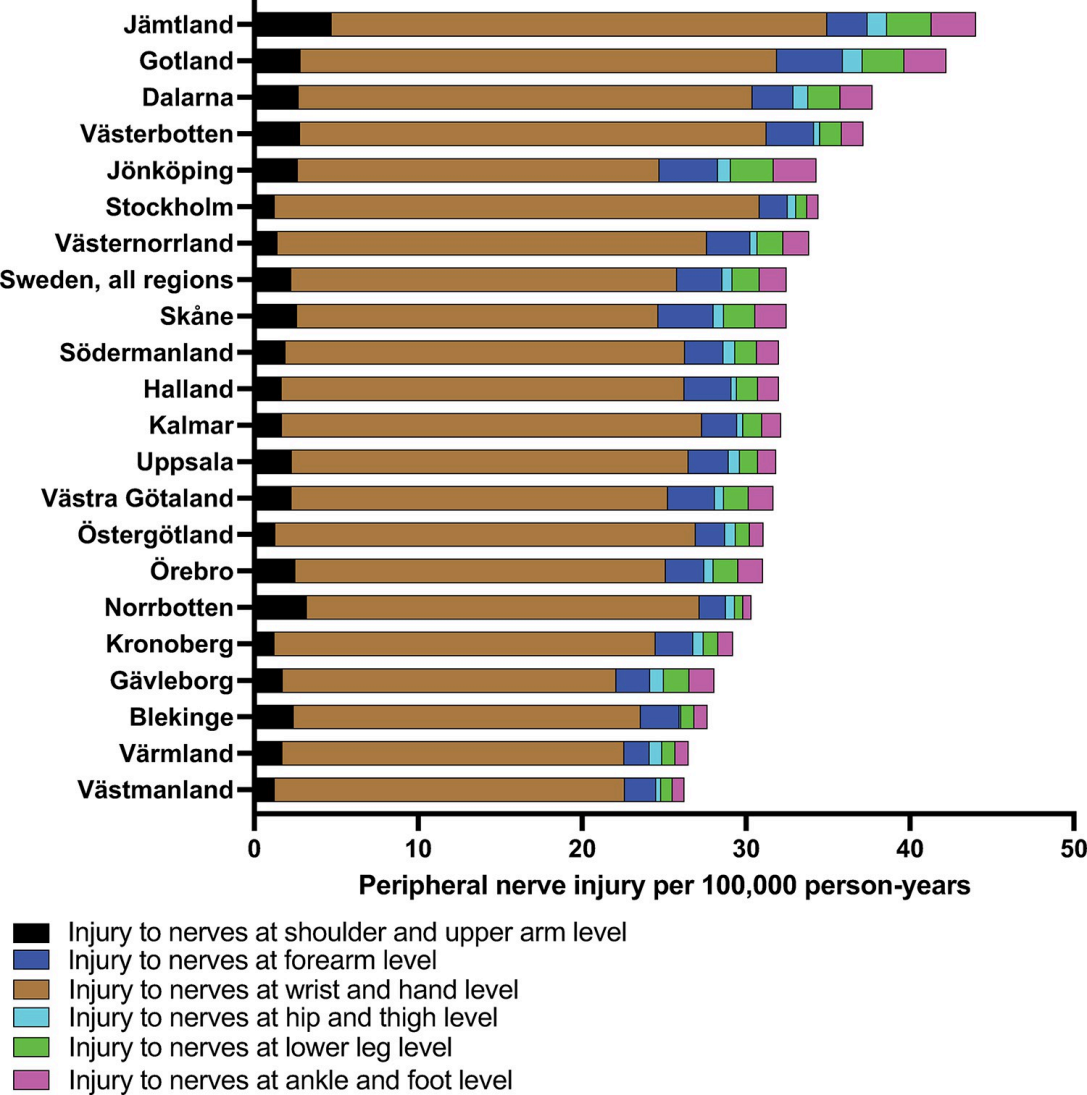
Regional variance in peripheral nerve injuries. Injuries are categorized per region and anatomical location.

The regions of Jämtland (p<0.001), Gotland (p = 0.004), Dalarna (p = 0.012), and Västerbotten (p = 0.032), show higher rates of peripheral nerve injuries while the regions of Värmland (p = 0.007) and Västmanland (p = 0.009), showed lower rates compared to the national average. There was also variance in the distribution of peripheral injuries in some regions. Jämtland for example, showed high rates of peripheral nerve injuries across various locations but also relatively higher rates in the upper extremities, such as the shoulder and upper arm (Fig 4).

**Fig 5:**
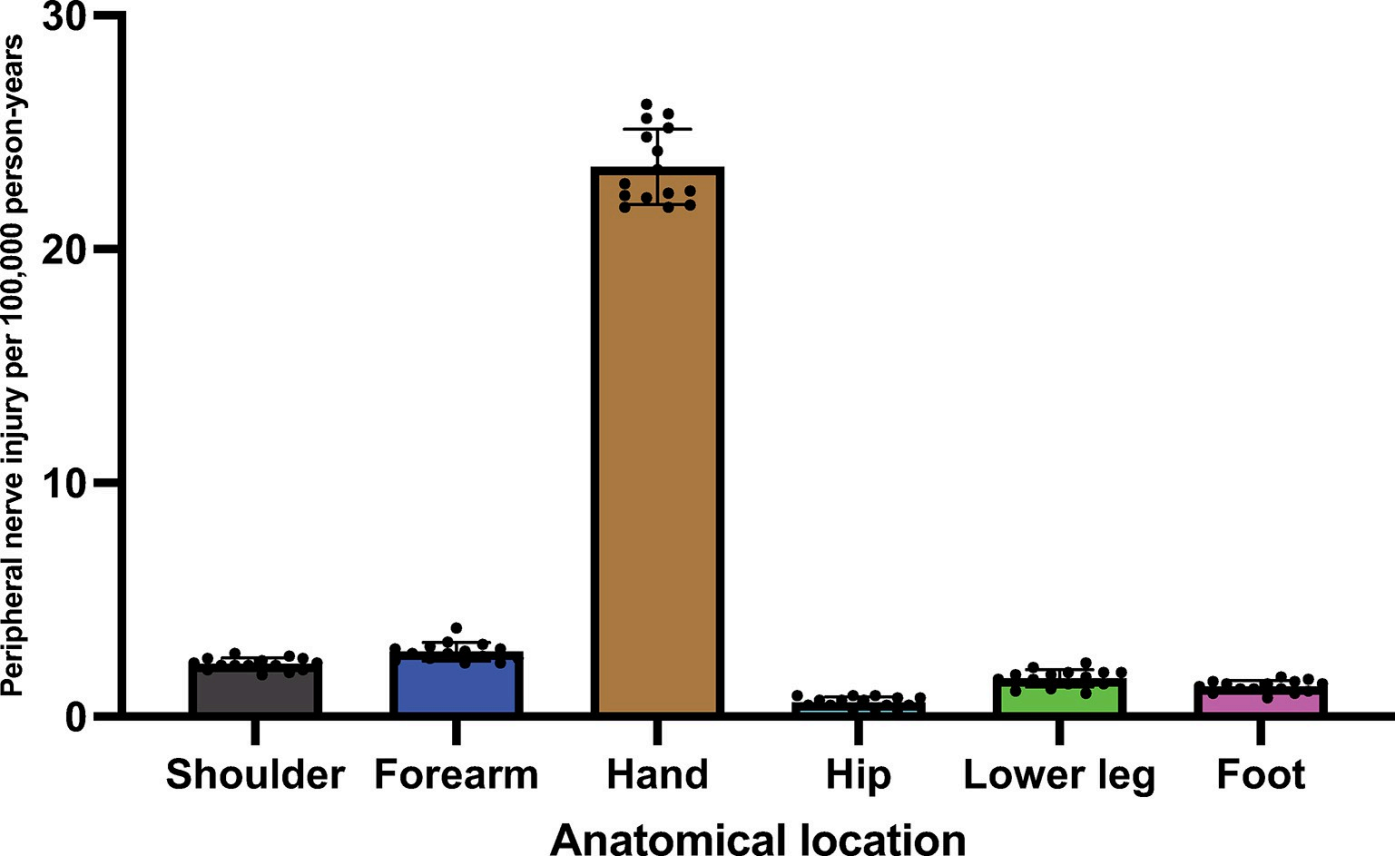
Distribution of peripheral nerve injuries across anatomical sites. Injury distribution during 2008–2022. Dots indicate years, error bars indicate 95% confidence interval.

Peripheral nerve injuries are the hand and wrist level were significantly more common than other categories (p = 0.4 x 10^−3^) (Fig 5).

## Discussion

When considering treatment of central nervous system disorders, specialized inpatient rehabilitation services are acknowledged for their cost-effective capacity in rehabilitating working-age patients [17]. However, there remains uncertainty regarding the extent of rehabilitation services available nationwide for patients with peripheral nerve injuries, indicating a need for comprehensive studies of the epidemiology and regional variance of traumatic peripheral nerve injuries.

Our observed incidence of peripheral nerve injuries, 15.6 per 100,000 in men and 10.1 per 100,000 in women, aligns closely with findings from other studies. A Finnish study reported similar rates for upper limb injuries specifically, while an English study, despite using slightly different ICD-10 codes that included unspecified nerve injury levels, also yielded comparable results. [18,19]. However, unlike England and Finland, Sweden displays a trend of decreasing peripheral nerve injuries during the study period which is a novel finding. A study from the U.S. by Karsy et al reported an incidence of 43.8 per million for upper extremity peripheral nerve injuries, higher than our overall rates of 15.6 per 100,000 in men and 10.1 per 100,000 in women for all peripheral nerve injuries. Both our study and Karsy et al observed a decreasing trend in PNIs over time [20]. A study by Padovano et al found a high incidence of peripheral nerve injuries following trauma, particularly in the upper extremities, similar to our findings [21]. Consistent with existing literature, our findings show a higher incidence of peripheral nerve injuries among men compared to women. This sex-based disparity is most likely explained due to underlying factors such as occupational hazards, anatomical differences, and behavioral patterns [18,22].

Our study shows a decreasing trend in the incidence of traumatic peripheral nerve injuries over the study period, with notable regional variations. It’s important to consider that the COVID-19 pandemic may have influenced these trends, particularly in the later years of our analysis. During the pandemic, patients with less severe injuries might have been reluctant to seek care, or their care may have been delayed due to healthcare system strain. This could have led to an underreporting of peripheral nerve injuries during this period, affecting the overall incidence rates. Future studies should investigate the pandemic’s impact on injury reporting and healthcare-seeking behavior to provide a clearer understanding of these trends.

The age-stratified analysis revealed a consistent trend of higher injury rates among males across all age groups. The most common group to sustain traumatic peripheral nerve injuries are men between 15 and 60 years of age. This is slightly older than what have been shown in previous studies [18,19]. These results raise questions about how to protect the elderly population against traumatic peripheral nerve injuries as these are associated with higher comorbidity and mortality in the elderly [23]. Efforts to promote safety awareness, implement age-appropriate rehabilitation protocols, and integrate ergonomic principles into occupational settings can help mitigate the burden of peripheral nerve injuries in the elderly. This is especially important as recovery after peripheral nerve injury is associated with increased disability compared to younger populations [24].

Our findings can help guide clinical discourse. Age and sex related differences help clinicians identify patient populations ar risk of injury while the detailed regional data provided in the study can assist clinicians in understanding the local epidemiology of peripheral nerve injuries. Clinicians can focus on early intervention and targeted rehabilitation or injury prevention counseling during routine check-ups, particularly for hand and wrist injuries, which are most prevalent. Public health officials can develop region-specific strategies based on the identified regional variance in injury rates, ensuring that resources and preventive measures are directed where they are most needed. This might include enhancing workplace safety regulations in high-incidence areas, healthcare accessability and increasing public awareness about injury prevention. Regional variance in injury prevlanece also allows for more informed decision-making when considering resource allocation, such as the need for specialized care units or additional training for healthcare professionals in regions with higher incidence rates.

The anatomical distribution of peripheral nerve injuries revealed distinct patterns, with the hand and wrist emerging as the most commonly affected sites. The higher incidence of peripheral nerve injuries in the forearm and hand can reasonably be attributed to the superficial anatomical positioning of the nerves in these locations. This also aligns with the functional importance and susceptibility of these regions to trauma, given their involvement in various activities of daily living and occupational tasks. These results are also mirrored by studies from both England and Finland which show significantly higher proportion of upper limb nerve injuries [18,19]. We were unable to determine the cause of peripheral injuries due to the nature our dataset, but other studies have found that upper limb nerve injuries are associated with trauma and occupational hazards [25,26]. Furthermore, distal upper limb nerve injuries in the elderly can be associated to falls, which could explain why we found a relatively high incidence of peripheral nerve injuries even in older groups [27,28].

Regional variations in the incidence of peripheral nerve injuries were evident, with certain regions consistently exhibiting higher rates compared to others. Some regions in particular exhibited larger proportion of upper limb injuries compared to others. This is important as wrist and upper limb peripheral nerve injuries are associated with significantly reduced limb function and pain, placing an uneven healthcare burden on some regions [29,30]. This is a previously unreported finding and warrant further investigation. Geographical differences on a national level could be explained by environmental conditions, access to healthcare services, and cultural practices in healthcare delivery [31–33]. In Sweden, specialized hand surgical units, which are crucial for managing injuries to large nerve trunks in the upper extremity, are not uniformly distributed across all regions. Regions without such specialized facilities often refer patients to other regions where these services are available. This referral pattern could lead to an apparent increase in the incidence of reported peripheral nerve injuries in regions with specialized clinics, as they not only manage local cases but also receive cases from neighboring regions lacking such facilities. Moreover, plexus injuries are nationally centralized to Umeå(Region Västerbotten), which could explain the higher incidence of these specific injuries in that region. The centralization of plexus injury treatment means that patients from across the country are referred to Umeå for specialized care, potentially inflating the incidence rates for this region compared to other. The identification of region specific challenges and etiology of peripheral nerve injuries could prove valuable in guiding public health interventions, and regional policy initiatives. Upper limb nerve injuries in particular are associated with significant healthcare costs [34].

The strengths of our study include its nationwide scope, long-term analysis, and utilization of high-quality register-based data from the NPR. By leveraging population-level data, we provide robust epidemiological insights into peripheral nerve injuries. However, certain limitations should be acknowledged. There is a potential for underreporting and misclassification in the data, which could impact the accuracy of the findings. Underreporting might lead to an underestimation of the true incidence, while misclassification could skew the results regarding the most affected demographics and injury sites. Due to the nature of our data, underreporting is likely to be low while misclassification could be a larger issue. To address these issues in future research, enhancing data validation through cross-referencing with other health records, such as regional internal quality registers, and improving the accuracy of ICD-10 coding are essential steps. Incorporating patient-level data could also provide deeper insights into the causes and outcomes of these injuries, identifying comorbidities and lead to more effective interventions. Within this study, we were unable to analyze individual patient-level data. However, such an analysis would facilitate more extensive comparisons between epidemiological subgroups.

## Conclusion

In conclusion, our study highlights the epidemiology of traumatic peripheral nerve injuries in Sweden over a time period of 15 years. We found a significant decrease in injuries, particularly in the hand and wrist, with disparities in rates by sex and age. Regional variations suggest the need for targeted interventions. While our study provides valuable insights, limitations such as underreporting should be considered. Further research incorporating individual patient-level data could enhance our understanding and inform preventive strategies.

## References

[1] Lee SK, Wolfe SW. Peripheral nerve injury and repair. J Am Acad Orthop Surg. 2000;8: 243–252. doi: 10.5435/00124635-200007000-00005 10951113

[2] Asplund M, Nilsson M, Jacobsson A, von Holst H. Incidence of traumatic peripheral nerve injuries and amputations in Sweden between 1998 and 2006. Neuroepidemiology. 2009;32: 217–228. doi: 10.1159/000197900 19174611

[3] Menorca RMG, Fussell TS, Elfar JC. Peripheral Nerve Trauma: Mechanisms of Injury and Recovery. Hand Clin. 2013;29: 317–330. doi: 10.1016/j.hcl.2013.04.002 23895713 PMC4408553

[4] Liu X, Duan X. Mechanisms and Treatments of Peripheral Nerve Injury. Ann Plast Surg. 2023;91: 313–318. doi: 10.1097/SAP.0000000000003480 36880740

[5] Eser F, Aktekin LA, Bodur H, Atan C. Etiological factors of traumatic peripheral nerve injuries. Neurol India. 2009;57: 434–437. doi: 10.4103/0028-3886.55614 19770544

[6] Wang ML, Rivlin M, Graham JG, Beredjiklian PK. Peripheral nerve injury, scarring, and recovery. Connect Tissue Res. 2019;60: 3–9. doi: 10.1080/03008207.2018.1489381 30187777

[7] Bergmeister KD, Große-Hartlage L, Daeschler SC, Rhodius P, Böcker A, Beyersdorff M, et al. Acute and long-term costs of 268 peripheral nerve injuries in the upper extremity. PLoS One. 2020;15: e0229530. doi: 10.1371/journal.pone.0229530 32251479 PMC7135060

[8] Li R, Liu Z, Pan Y, Chen L, Zhang Z, Lu L. Peripheral nerve injuries treatment: a systematic review. Cell Biochem Biophys. 2014;68: 449–454. doi: 10.1007/s12013-013-9742-1 24037713

[9] Lopes B, Sousa P, Alvites R, Branquinho M, Sousa AC, Mendonça C, et al. Peripheral Nerve Injury Treatments and Advances: One Health Perspective. Int J Mol Sci. 2022;23: 918. doi: 10.3390/ijms23020918 35055104 PMC8779751

[10] Ehni BL. Treatment of traumatic peripheral nerve injury. Am Fam Physician. 1991;43: 897–905. 2000734

[11] Robinson MD, Shannon S. Rehabilitation of peripheral nerve injuries. Phys Med Rehabil Clin N Am. 2002;13: 109–135. doi: 10.1016/s1047-9651(03)00074-3 11878078

[12] Statistikdatabas. In: Socialstyrelsen [Internet]. 15 Jan 2021 [cited 8 Jun 2023]. Available: https://sdb.socialstyrelsen.se/if_ope/val.aspx.

[13] Statistics Sweden. Statistical databas. Available: https://www.statistikdatabasen.scb.se/pxweb/en/ssd/START__BE__BE0101/.

[14] Benchimol EI, Smeeth L, Guttmann A, Harron K, Moher D, Petersen I, et al. The REporting of studies Conducted using Observational Routinely-collected health Data (RECORD) statement. PLoS Med. 2015;12: e1001885. doi: 10.1371/journal.pmed.1001885 26440803 PMC4595218

[15] Steindel SJ. International classification of diseases, 10th edition, clinical modification and procedure coding system: descriptive overview of the next generation HIPAA code sets. J Am Med Inform Assoc. 2010;17: 274–282. doi: 10.1136/jamia.2009.001230 20442144 PMC2995704

[16] Ludvigsson JF, Andersson E, Ekbom A, Feychting M, Kim J-L, Reuterwall C, et al. External review and validation of the Swedish national inpatient register. BMC Public Health. 2011;11: 450. doi: 10.1186/1471-2458-11-450 21658213 PMC3142234

[17] Turner-Stokes L, Williams H, Bill A, Bassett P, Sephton K. Cost-efficiency of specialist inpatient rehabilitation for working-aged adults with complex neurological disabilities: a multicentre cohort analysis of a national clinical data set. BMJ Open. 2016;6: e010238. doi: 10.1136/bmjopen-2015-010238 26911586 PMC4769383

[18] Wiman K, Hulkkonen S, Miettunen J, Auvinen J, Karppinen J, Ryhänen J. Total, gender- and age-specific incidence rates of upper extremity nerve injuries in Finland. J Hand Surg Eur Vol. 2022;47: 639–643. doi: 10.1177/17531934221079230 35172640

[19] Murphy RNA, de Schoulepnikoff C, Chen JHC, Columb MO, Bedford J, Wong JK, et al. The incidence and management of peripheral nerve injury in England (2005–2020). Journal of Plastic, Reconstructive & Aesthetic Surgery. 2023;80: 75–85. doi: 10.1016/j.bjps.2023.02.017 36996504

[20] Karsy M, Watkins R, Jensen MR, Guan J, Brock AA, Mahan MA. Trends and Cost Analysis of Upper Extremity Nerve Injury Using the National (Nationwide) Inpatient Sample. World Neurosurg. 2019;123: e488–e500. doi: 10.1016/j.wneu.2018.11.192 30502477

[21] Padovano WM, Dengler J, Patterson MM, Yee A, Snyder-Warwick AK, Wood MD, et al. Incidence of Nerve Injury After Extremity Trauma in the United States. Hand (New York, N,Y). 2022;17: 615–623. doi: 10.1177/1558944720963895 33084377 PMC9274890

[22] Miranda GE, Torres RY. Epidemiology of Traumatic Peripheral Nerve Injuries Evaluated with Electrodiagnostic Studies in a Tertiary Care Hospital Clinic. P R Health Sci J. 2016;35: 76–80. 27232868

[23] Novak CB, Anastakis DJ, Beaton DE, Mackinnon SE, Katz J. Biomedical and psychosocial factors associated with disability after peripheral nerve injury. J Bone Joint Surg Am. 2011;93: 929–936. doi: 10.2106/JBJS.J.00110 21593368

[24] Andrasfay T, Raymo N, Goldman N, Pebley AR. Physical work conditions and disparities in later life functioning: Potential pathways. SSM Popul Health. 2021;16: 100990. doi: 10.1016/j.ssmph.2021.100990 34917747 PMC8666356

[25] Silver S, Ledford CC, Vogel KJ, Arnold JJ. Peripheral Nerve Entrapment and Injury in the Upper Extremity. Am Fam Physician. 2021;103: 275–285. 33630556

[26] Tapp M, Wenzinger E, Tarabishy S, Ricci J, Herrera FA. The Epidemiology of Upper Extremity Nerve Injuries and Associated Cost in the US Emergency Departments. Ann Plast Surg. 2019;83: 676–680. doi: 10.1097/SAP.0000000000002083 31688105

[27] Bekelis K, Missios S, Spinner RJ. Falls and peripheral nerve injuries: an age-dependent relationship. J Neurosurg. 2015;123: 1223–1229. doi: 10.3171/2014.11.JNS142111 25978715

[28] Missios S, Bekelis K, Spinner RJ. Traumatic peripheral nerve injuries in children: epidemiology and socioeconomics. J Neurosurg Pediatr. 2014;14: 688–694. doi: 10.3171/2014.8.PEDS14112 25303155

[29] Irwin MS, Gilbert SE, Terenghi G, Smith RW, Green CJ. Cold intolerance following peripheral nerve injury. Natural history and factors predicting severity of symptoms. J Hand Surg Br. 1997;22: 308–316. doi: 10.1016/s0266-7681(97)80392-0 9222907

[30] Rosberg H-E, Carlsson KS, Dahlin LB. Prospective study of patients with injuries to the hand and forearm: costs, function, and general health. Scand J Plast Reconstr Surg Hand Surg. 2005;39: 360–369. doi: 10.1080/02844310500340046 16298809

[31] Baum A, Wisnivesky J, Basu S, Siu AL, Schwartz MD. Association of Geographic Differences in Prevalence of Uncontrolled Chronic Conditions With Changes in Individuals’ Likelihood of Uncontrolled Chronic Conditions. JAMA. 2020;324: 1429–1438. doi: 10.1001/jama.2020.14381 33048153 PMC8094427

[32] Volinn E, Diehr P, Ciol MA, Loeser JD. Why does geographic variation in health care practices matter? (And seven questions to ask in evaluating studies on geographic variation). Spine (Phila Pa 1976). 1994;19: 2092S–2100S. doi: 10.1097/00007632-199409151-00012 7801188

[33] Mulyanto J, Kunst AE, Kringos DS. Geographical inequalities in healthcare utilisation and the contribution of compositional factors: A multilevel analysis of 497 districts in Indonesia. Health Place. 2019;60: 102236. doi: 10.1016/j.healthplace.2019.102236 31778844

[34] Dy CJ, Lingampalli N, Peacock K, Olsen MA, Ray WZ, Brogan DM. Direct Cost of Surgically Treated Adult Traumatic Brachial Plexus Injuries. J Hand Surg Glob Online. 2020;2: 77–79. doi: 10.1016/j.jhsg.2019.12.001 32864587 PMC7454232

